# Correction: IMDB Network Revisited: Unveiling Fractal and Modular Properties from a Typical Small-World Network

**DOI:** 10.1371/annotation/7ce29312-158e-49b2-b530-6aca07751cea

**Published:** 2013-08-22

**Authors:** Lazaros K. Gallos, Fabricio Q. Potiguar, José S. Andrade, Hernan A. Makse

There were errors in the second and third institutions that authors are affiliated with. The correct versions are, respectively:

Faculdade de Física, ICEN, Universidade Federal do Pará, Belém, Pará, Brasil,

Departamento de Física, Universidade Federal do Ceará, Fortaleza, Ceará, Brasil

There was also an error in the Funding statement.

The correct statement is: The authors acknowledge support from Brazilian agencies CNPq, CAPES, and FUNCAP, the FUNCAP/Cnpq Pronex grant, PROPESP/UFPa, FADESP, and the National Institute of Science and Technology for Complex Systems in Brazil for financial support. The funders had no role in study design, data collection and analysis, decision to publish, or preparation of the manuscript. 

